# Holding the Secret: A Rare Case of Nausea and Vomiting Due to Ligamentous Compression of the Celiac Axis

**DOI:** 10.7759/cureus.9726

**Published:** 2020-08-13

**Authors:** Farhan Ali, Sowmya Srinivas, Tenzin Tseky, Hafiz Muzaffar Akbar Khan, Dayakar Reddy

**Affiliations:** 1 Internal Medicine, Arnot Ogden Medical Center, Elmira, USA; 2 Gastroenterology and Hepatology, Guthrie Robert Packer Hospital, Sayre, USA

**Keywords:** dunbar syndrome

## Abstract

Dunbar syndrome also known as median arcuate ligament syndrome (MALS) or celiac artery compression syndrome (CACS) is a rare syndrome resulting from the external compression of the celiac trunk from the median arcuate ligament. A 78-year-old female with multiple chronic conditions presented with intermittent, post-prandial epigastric pain associated with early satiety, decreased appetite for òne year. Multiple tests including gastric emptying scan and hepatobiliary scan with cholecystokinin (CCK) were normal. Contrast-enhanced computed tomography (CECT) abdomen/pelvis showed thickening of a median arcuate ligament. Further imaging with end-inspiratory phase computed tomography (CT) angiography of the abdomen and 3D reconstruction of images, revealed approximately 1 cm length segment of proximal celiac arterial narrowing, measuring 70% maximally (at its origin) and characteristic hooked appearance of the proximal celiac artery with post-stenotic dilation diagnostic of MALS. Our case report emphasizes the importance of MALS in the differential diagnosis of chronic, intermittent abdominal pain.

## Introduction

Dunbar Syndrome, also known as Median Arcuate Ligament Syndrome (MALS), was first described by Harjola in 1963 and Dunbar et al. in 1965 [1,2]. It is a rare condition with a reported incidence of 2 per 100,000 more commonly noted in women aged 30-50 years and in thin patients [3,4]. We present a case of 78-year-old female who presented to the hospital for persistent abdominal pain of unknown etiology, who was later diagnosed with Dunbar syndrome after being found on CT to have extrinsic compression of the proximal celiac axis.

## Case presentation

We present a case a 78-year-old Caucasian female with a past medical history notable for hypertension, hyperlipidemia, hypothyroidism, chronic obstructive pulmonary disease (COPD), irritable bowel syndrome, diverticulosis, hemorrhoids, two pre-pyloric gastric ulcers and hiatal hernia (biopsies negative for Helicobacter pylori), who presented to our facility with chief complaints of epigastric pain, weakness, persistent nausea with associated dry heaving and one episode of non-bloody, non-bilious emesis. The patient stated that her epigastric pain was intermittent and post-prandial in nature. She had developed progressive worsening of her symptoms over the course of one year which worsened in the past four months. She admitted to occasional pyrosis and attempted to treat her symptoms with her home regimen of omeprazole, and bismuth, with modest improvement in her dyspeptic symptoms. She had early satiety, a substantial reduction in appetite, and insomnia which she attributed to migraines. She admitted to using occasional medical marijuana in the past but denied recent use. She denied new medications, aside from taking fioricet for migraine headaches. She also had a 12-pound unintentional weight loss over the course of her symptom progression. She denied hematochezia, melena, night sweats, odynophagia or dysphagia. No history of recent non-steroidal anti-inflammatory drugs or steroid use. A detailed medication reconciliation was conducted and did not reveal any history of overt usage of gastrotoxic drugs. She was a former smoker and quit smoking 10 years ago. She denied alcohol or illicit drug use. Her family history was negative for any gastrointestinal malignancy.

On admission, the patient was alert and oriented. She was afebrile, with a heart rate of 103 beats per min, respiratory rate of 14, blood pressure was 168/82 and was saturating at 98% room air. Her abdomen was soft, non-distended, tenderness primarily in the mid epigastrium without guarding or rigidity. Normoactive bowel sounds in all four quadrants with no signs of hepatomegaly. Cardiac examination revealed II/VI systolic murmur at right second intercostal space. Trace edema was noted in lower extremities with intact pulses. The patient had no pallor, jaundice, lymphadenopathy. The respiratory and neurological examinations were normal.

Initial labs showed that she was hypokalemic with a potassium level of 3.2 mEq/L with no remarkable changes in her electrolytes, complete blood count and thyroid-stimulating hormone. Blood urea nitrogen and creatinine levels were 12 mg/dL and 1.2 mg/dL, respectively. Other laboratory workup included albumin 3.8 g/dL, total protein 7.1 g/dL, alkaline phosphatase 89 IU/L, total bilirubin 1.2 mg/dL, aspartate aminotransferase 21 U/L, alanine aminotransferase 20U/L and lipase 23 U/L. The patient had a normal gastric emptying scan and normal hepatobiliary scan with cholecystokinin (CCK).

**Figure 1:**
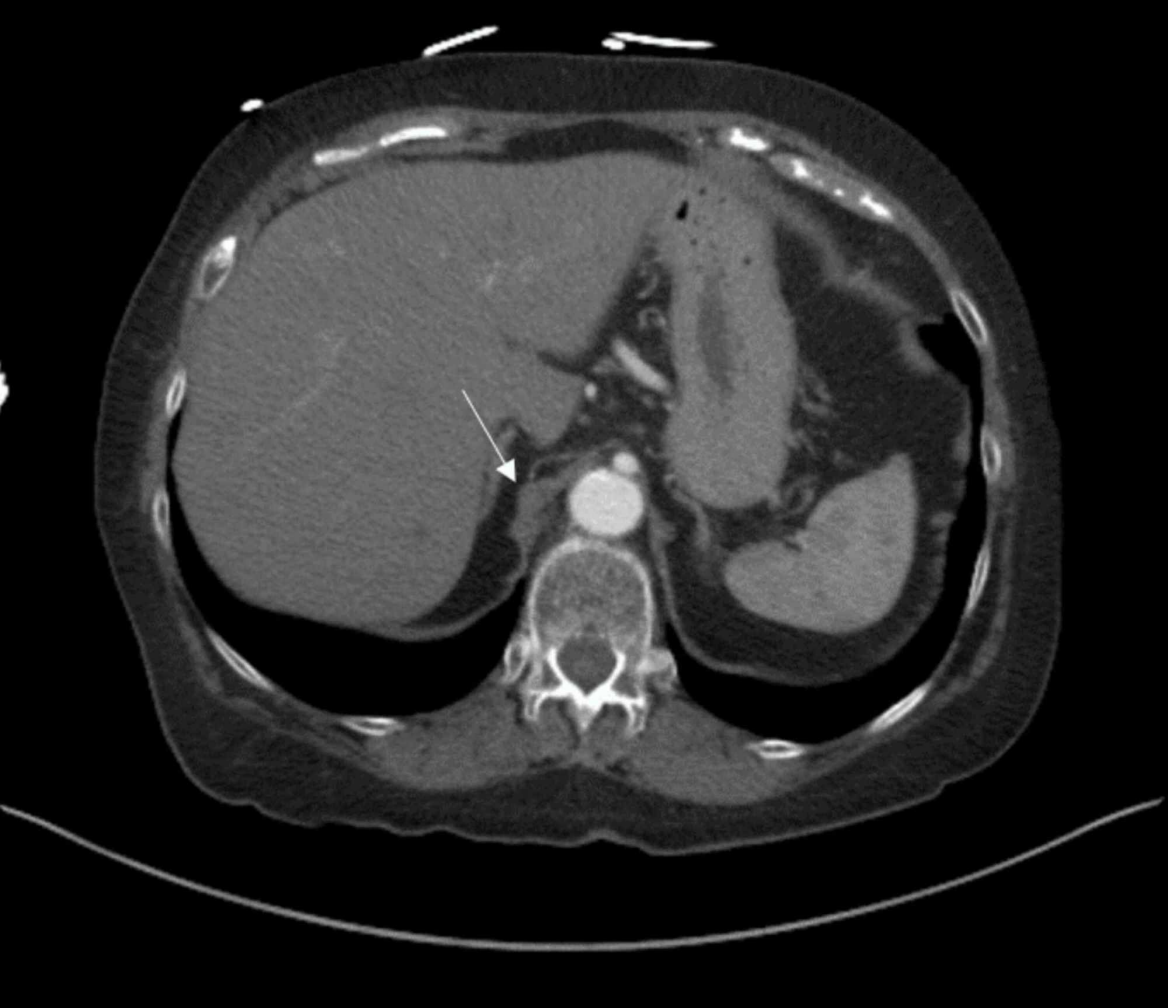
Axial contrast-enhanced CT image of abdomen/pelvis demonstrates the thickening of the median arcuate ligament on the right (arrow)

Initial imaging of the patient using CECT abdomen/pelvis showed external compression of celiac trunk due to the thickening of median arcuate ligament on the right (Figure 1).

**Figure 2:**
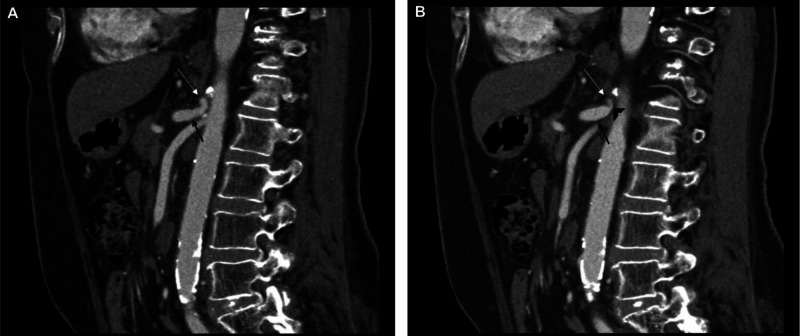
Sagittal CT angiography images of abdomen/pelvis (A, B) demonstrates proximal celiac artery narrowing (white arrows), post-stenotic dilation (black arrows), and (B) characteristic hooked appearance (arrowhead) of the proximal celiac artery

**Figure 3:**
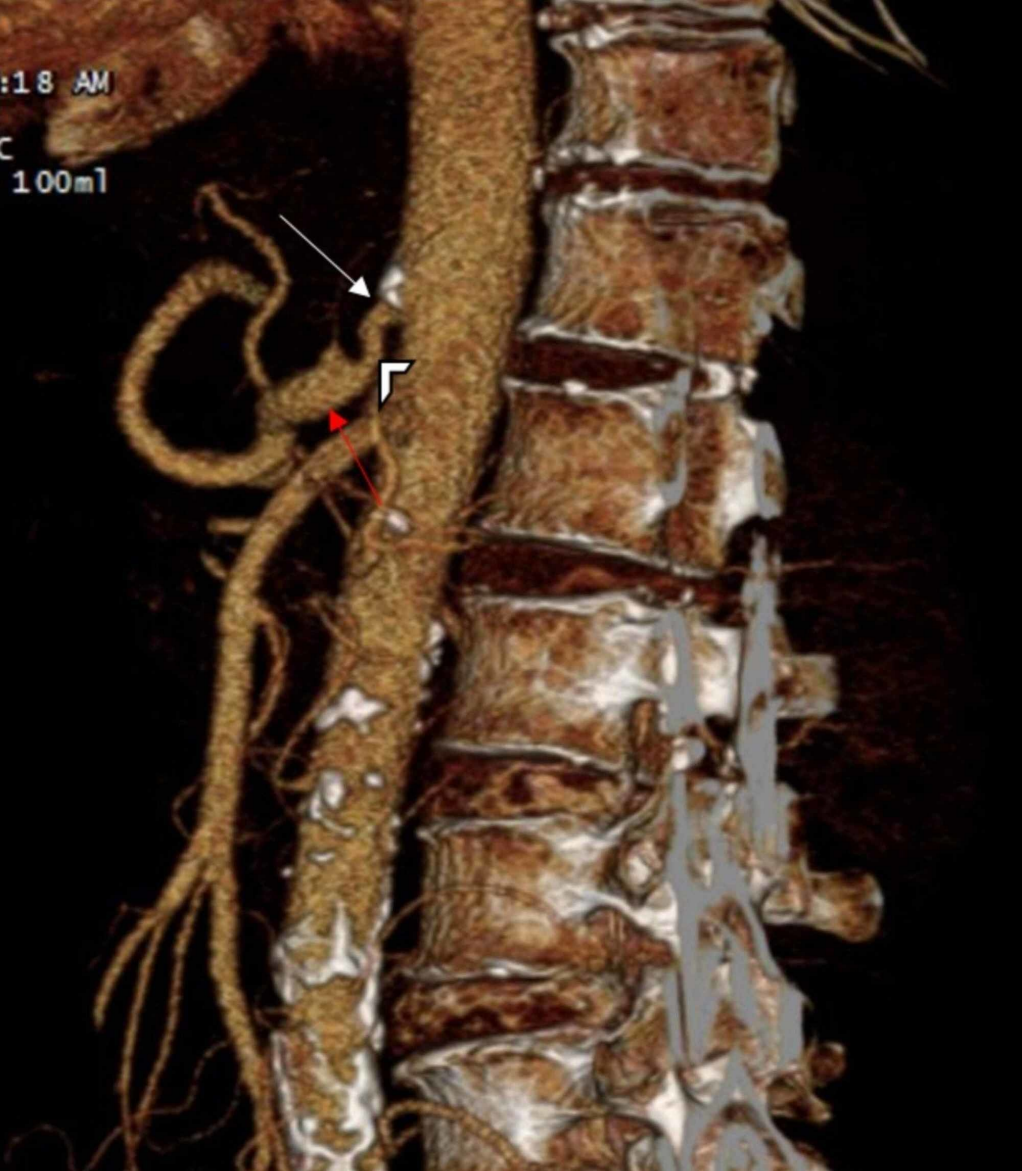
Sagittal 3D reconstruction image of abdomen/pelvis demonstrates the narrowing of proximal celiac artery (white arrow), characteristic hooked appearance of the proximal celiac artery (arrowhead) and post-stenotic dilation (red arrow)

Further diagnostic evaluation included utilizing end-inspiratory phase CT angiography of the abdomen and 3D reconstruction of images, which revealed an approximately 1 cm length segment of proximal celiac arterial narrowing, measuring 70% maximally (at its origin) and characteristic hooked appearance of the proximal celiac artery with post-stenotic dilation (Figures 2A, 2B, 3).

Vascular surgery was consulted, and upon review of the reconstruction of her CTA, moderate compression of her celiac artery was thus discovered, likely representing median arcuate ligament syndrome.

## Discussion

Dunbar syndrome results from the extrinsic compression of the celiac trunk by the median arcuate ligament. The median arcuate ligament forms a fibrous arch around the anterior aspect where the right and left crura join. The disease is marked by the hallmark feature of postprandial abdominal pain, as seen in other instances of mesenteric ischemia. Other signs and symptoms include nausea, vomiting, and consequent weight loss from inability to tolerate oral intake [5]. Up to 24% of patients with evidence of radiographic compression may present without any symptoms [6]. Symptoms are not only thought to result from mesenteric ischemia, but also thought to in part be a neurogenic disease, whereby the pain a patient feels originates from somatic nerves in the splanchnic plexus [6]. The patient was taking fiorcet for migraine headaches which is a drug with vasoconstrictor properties. Since the celiac trunk blood flow is already compromised by the ligament compression, the drug may have caused further decrease in the gut blood flow and worsened her symptoms.

Diagnosis is made using selective angiography, as the stenosis disappears during deep inspiration and reappears on expiration. Additional diagnostic modalities include gastric tonometry and percutaneous celiac ganglion block, doppler ultrasound, spiral CTA, magnetic angiography [3,7].

CT angiography and conventional angiography of the abdomen are the gold standard imaging modalities. These modalities demonstrate focal stenosis that has a characteristic hooked appearance due to the indentation of the celiac trunk on its superior surface. This characteristic hooked appearance of the stenosis, as well as the younger presenting age of the patient, distinguishes this syndrome from the main differential diagnosis of atherosclerotic disease.

It is important to note that superior indentation of the celiac trunk may be seen in normal people if imaging is acquired in expiration. Therefore, imaging for accurate diagnosis should ideally be performed in the end-inspiratory phase. Furthermore, imaging findings must also be correlated with the clinical history.

Additional features that may be appreciated include post-stenotic dilatation, prominent collaterals, such as the gastroduodenal and common hepatic arteries, and thickening of the median arcuate ligament. A thickness of the median arcuate ligament of greater than 4 mm is considered abnormal [8].

Treatment includes endovascular transluminal angioplasty and stent placement, as well as laparoscopic division of the arcuate ligament and resection of the celiac plexus [9,10]. Results following surgery may be varied. Many patients (up to 60%-70%) have successful resolution of symptoms however since post-operative pain may persist following intervention, pain may not resolve for several months following surgery [3,4].

## Conclusions

Dunbar syndrome is a rare clinical condition but should be considered in the differential diagnosis of unexplained gastrointestinal symptoms. CT and conventional angiography of the abdomen play an important role in the diagnosis of MALS. Some patients can be managed conservatively and show spontaneous resolution of symptoms. Definitive treatment consists of laparoscopic surgical intervention. In less common instances, stent placement may also be used.
